# Supplementary material for: Non-Antigenic Modulation of Antigen Receptor (TCR) Cβ-FG Loop Modulates Signalling: Implications of External Factors Influencing T-Cell Responses
Source: Int J Mol Sci. 2023 May 26;24(11):9334. doi: 10.3390/ijms24119334 (PMC10253556; doi:10.3390/ijms24119334)

Supplementary Figures:

Supplementary Figure 1. Structural models of TCR and binding of lead compounds. The molecular dynamics simulation predicted 5 compounds: **(A)** oestradiol, **(B)** N-acetyl-D-tryptophan, **(C)** D,L-homotryptophan, **(D)** malonate, and **(E)** nicotine that have the best score during docking analysis using CHIMERA. **(F)** Compounds including isobutyrate, 3-indole-butyrate and propanoate have no effect on T-cell activation due to either weak binding affinity or no interaction with FG loop. The TCR-αβ heterodimer complex and Cβ FG loop is coloured in beige and cyan respectively.

Supplementary Figure 2. Initial docking screening by PyRx.

Supplementary Figure 3. Docking poses of compounds which were found to either have no effect on, or reduce IL-2 production in the biological assays. The active compounds were found to cluster around the outside of the FG loop. The values underneath each image are the Prime MM-GBSA dG binding scores in (kcal/mol). Hydrogen bonds, salt bridges, π-π and cation-π interactions are denoted as dashed yellow, pink, blue and green lines respectively.

Supplementary Figure 4. The effect of compounds on the cell viability of immune hybridoma lines. The 2B4.11 and LK35.2 hybridoma lines were cultured for 24 h in the absence or presence of the following compounds at different concentrations: 3-indolebutyrate, isobutyrate, propanoate, malonate, nicotine, oestradiol, D,L-Homotryptophan, and N-Acetyl-D-tryptophan. All the data was expressed as mean ±SD, n = 3.

Supplementary Figure 1

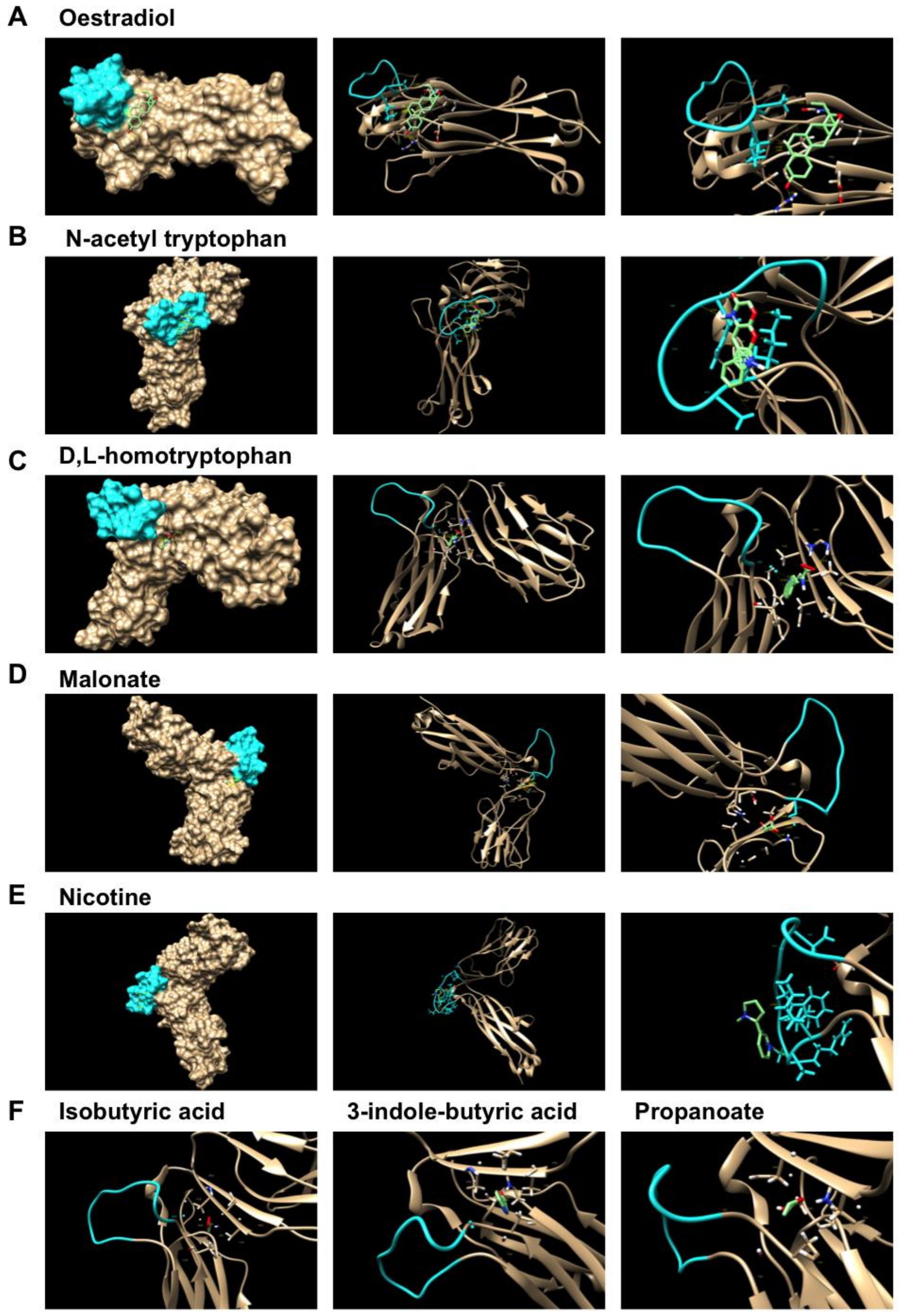

Supplementary Figure 2

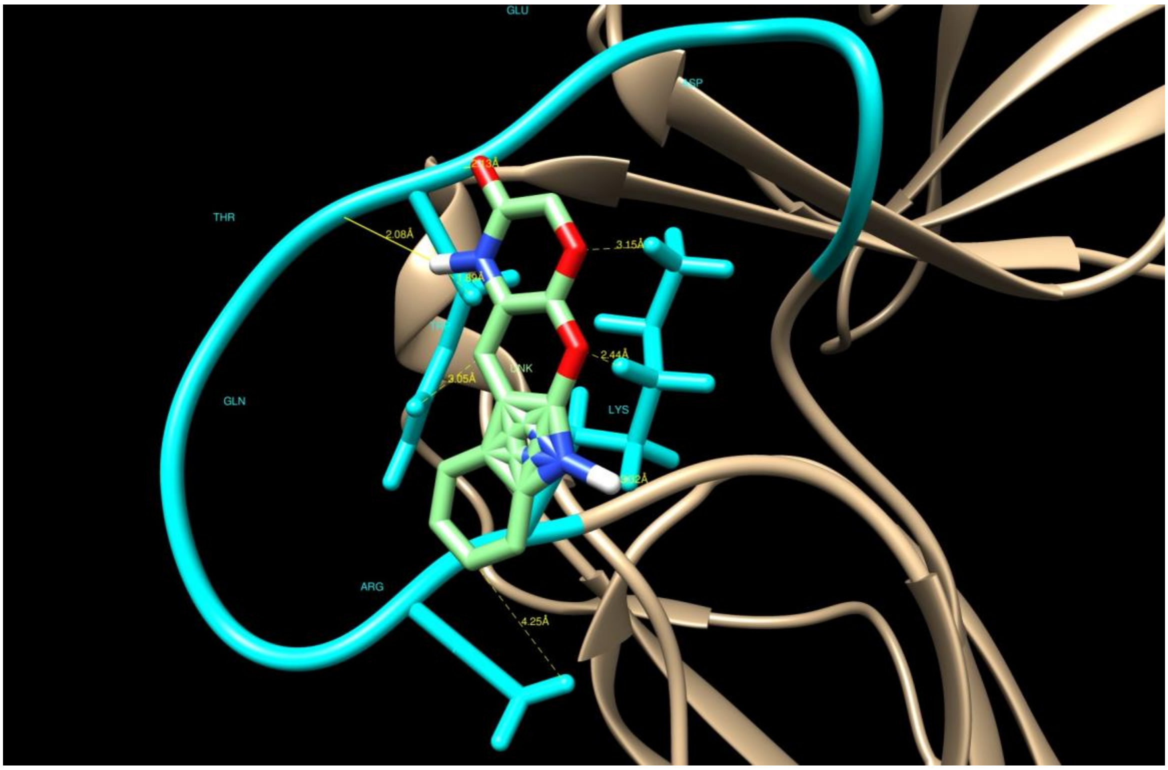

N-Acetyl-Tryptophan, showing distance of H-bond to FG loop residues (yellow line).

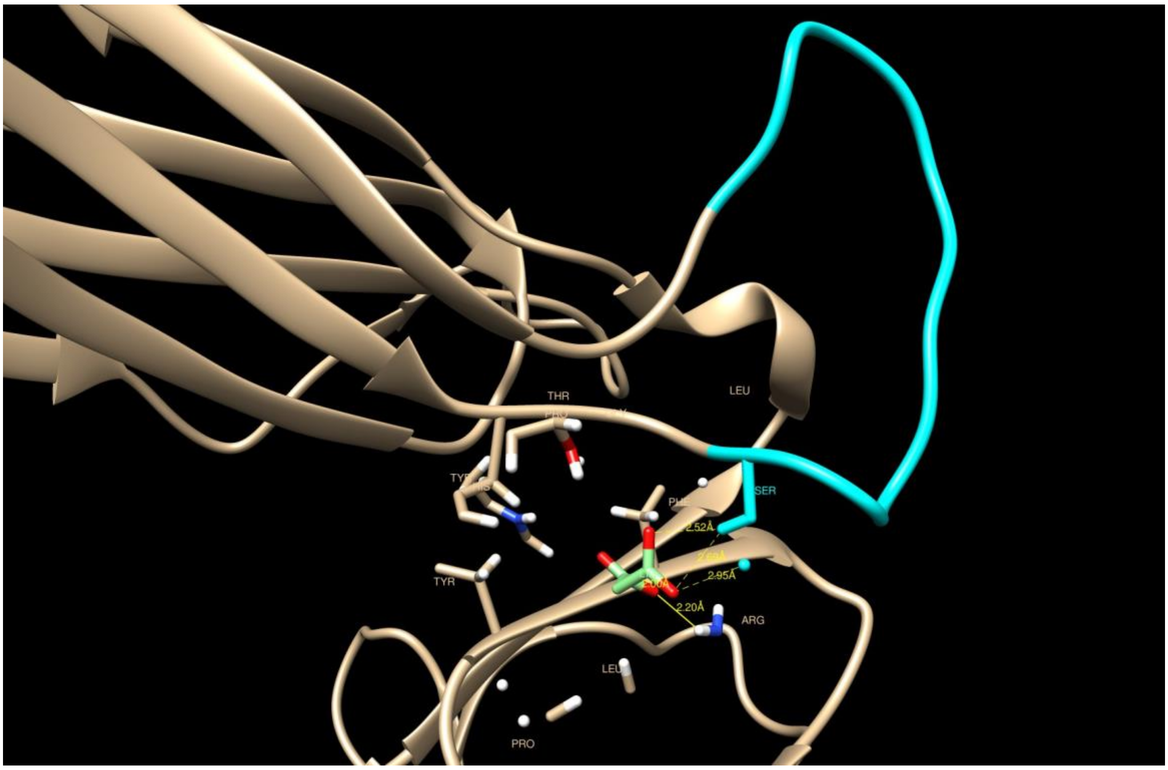

Malonic acid, showing distance of H-bond to FG loop residues (yellow line).

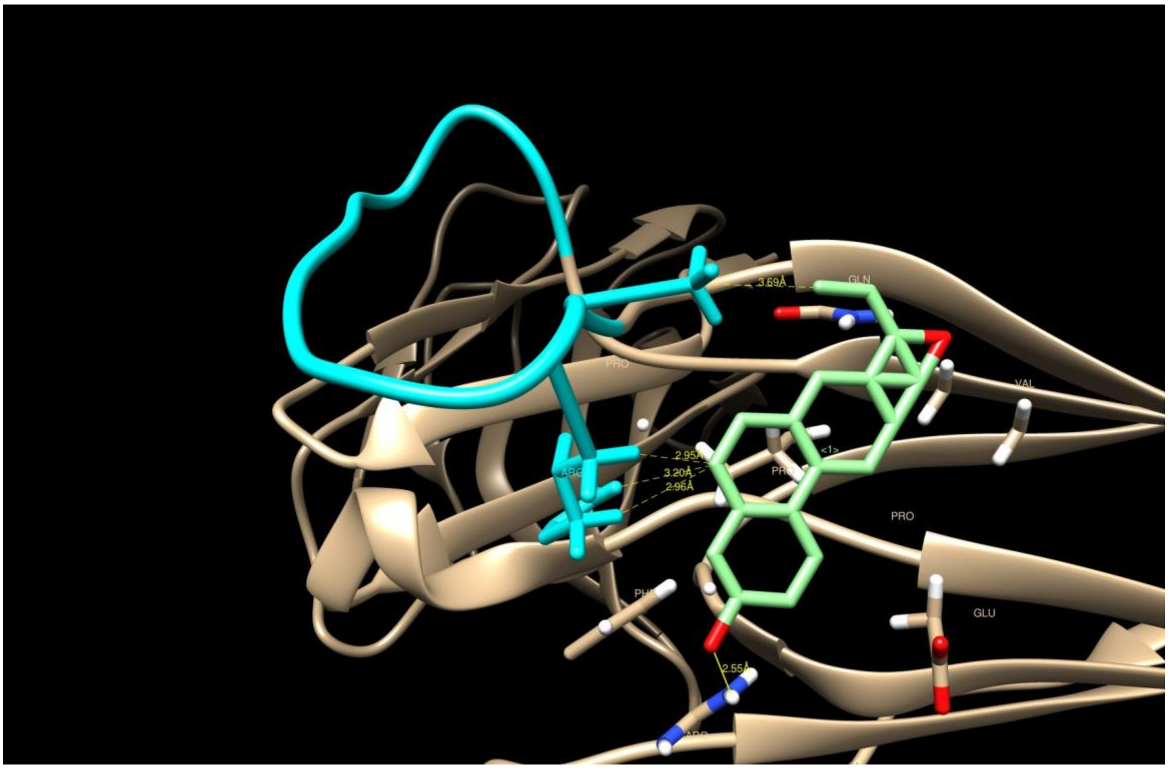

Oestradiol, showing distance of H-bond to FG loop residues (yellow line).

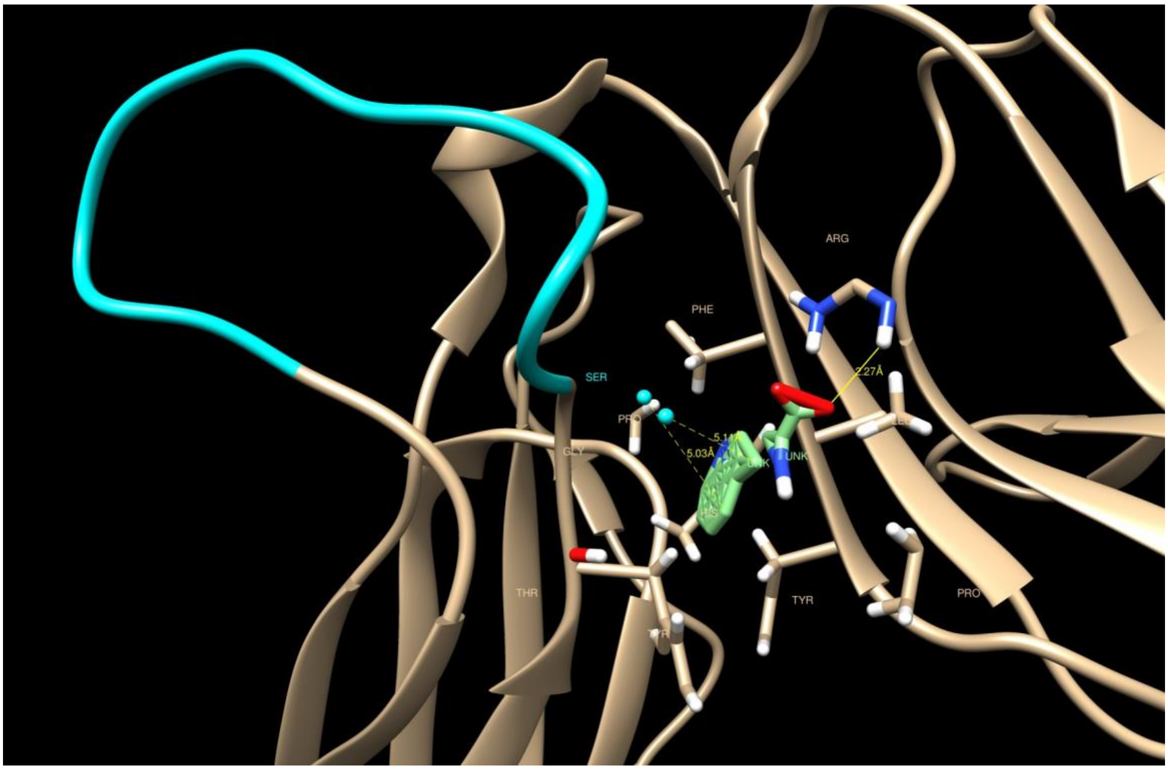

D,L-homotryptophan, showing distance of H-bond to FG loop residues (yellow line).

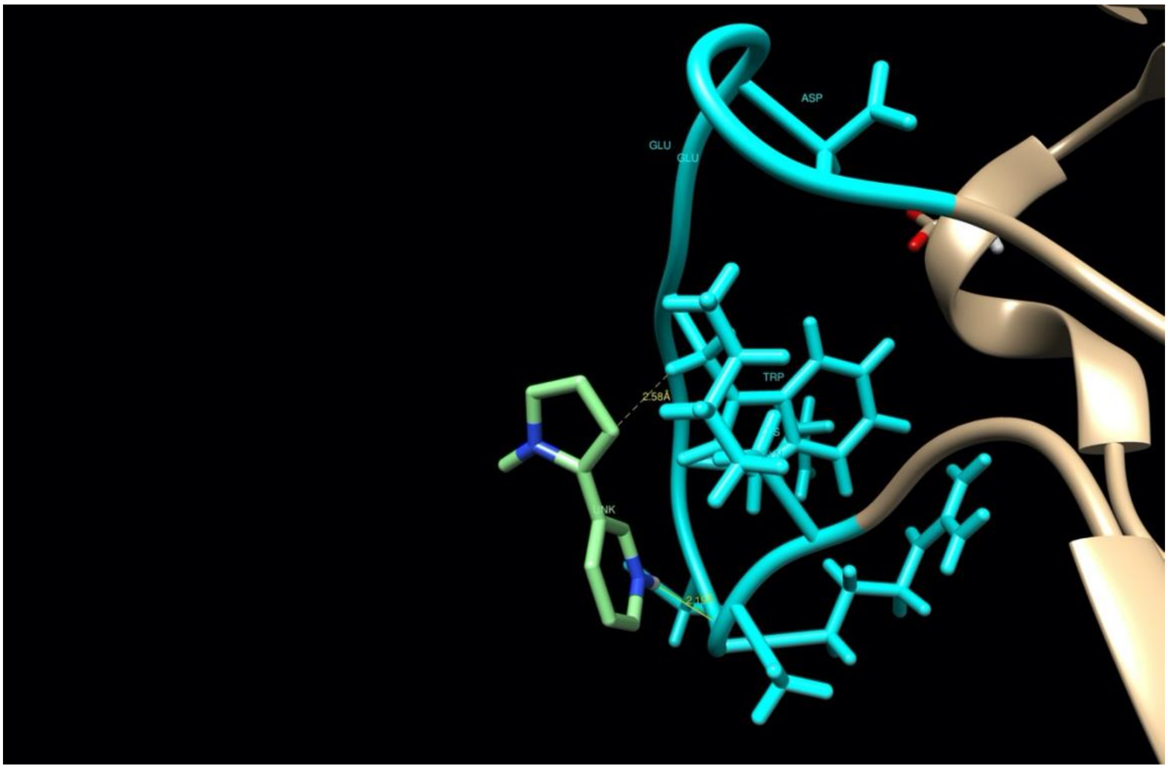

Nicotine, showing distance of H-bond to FG loop residues (yellow line).

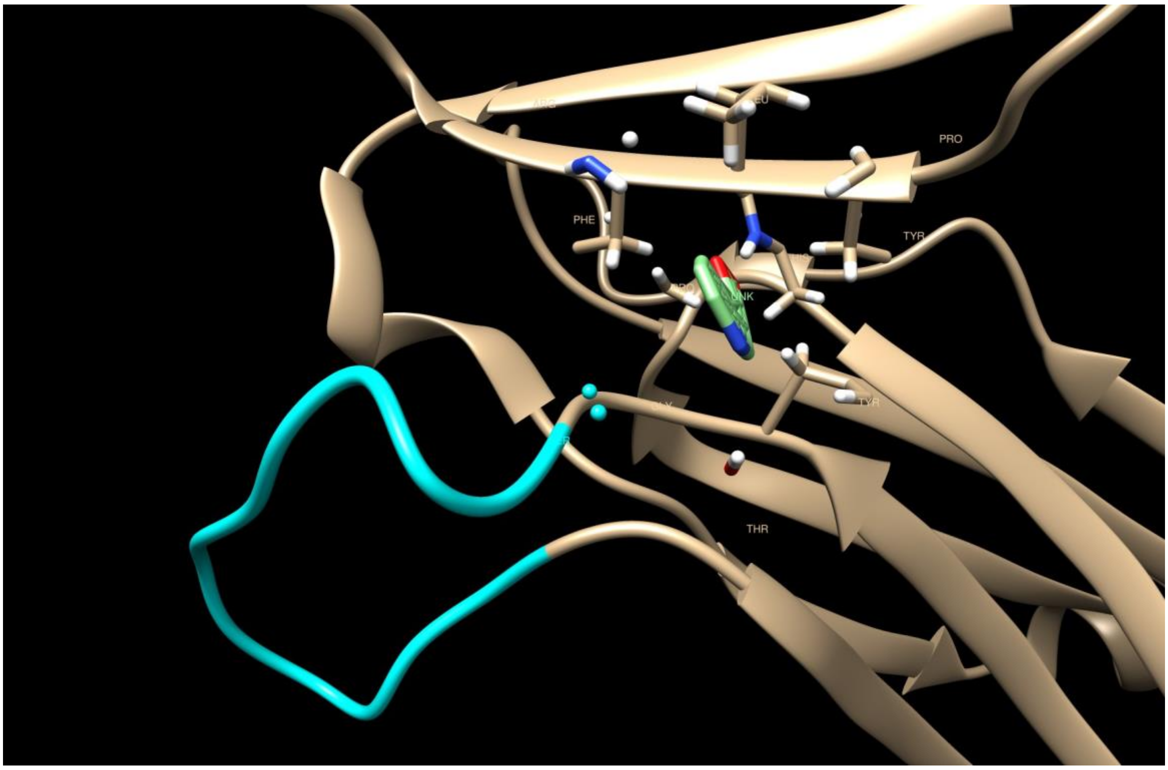

Indole-3-Butyric Acid, showing no H-bond to FG loop residues.

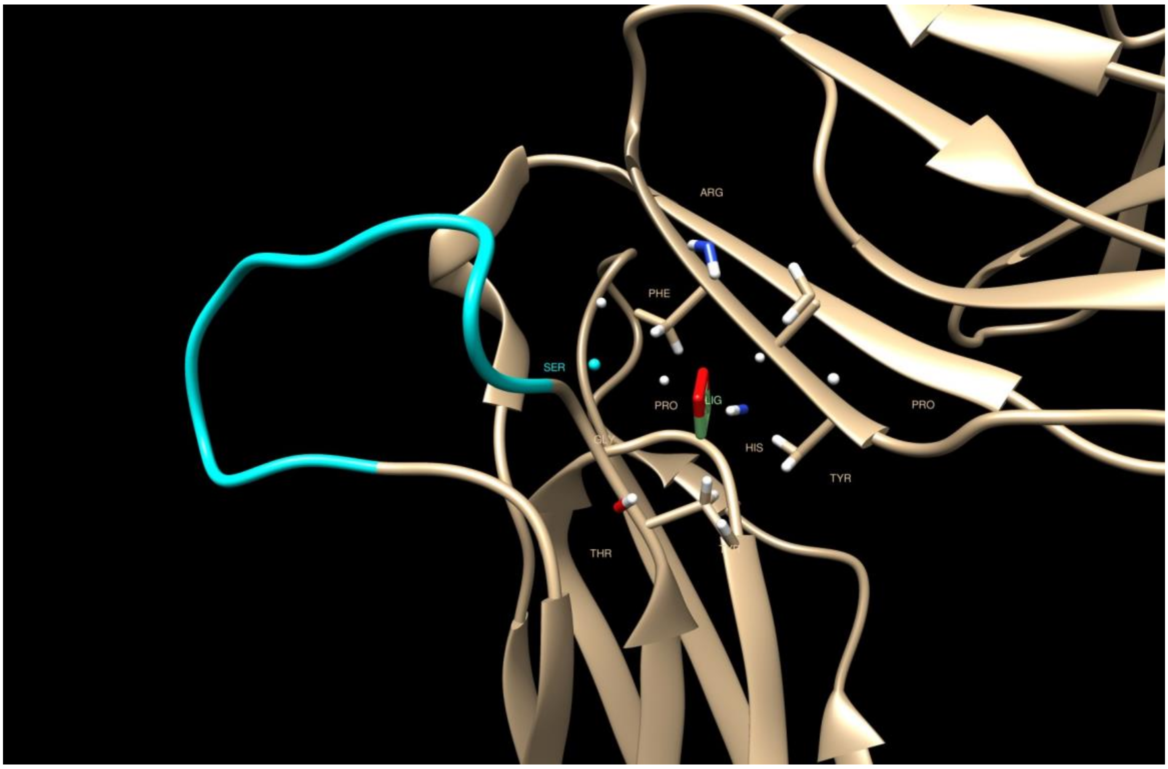

Isobutyric acid, showing no H-bond to FG loop residues.

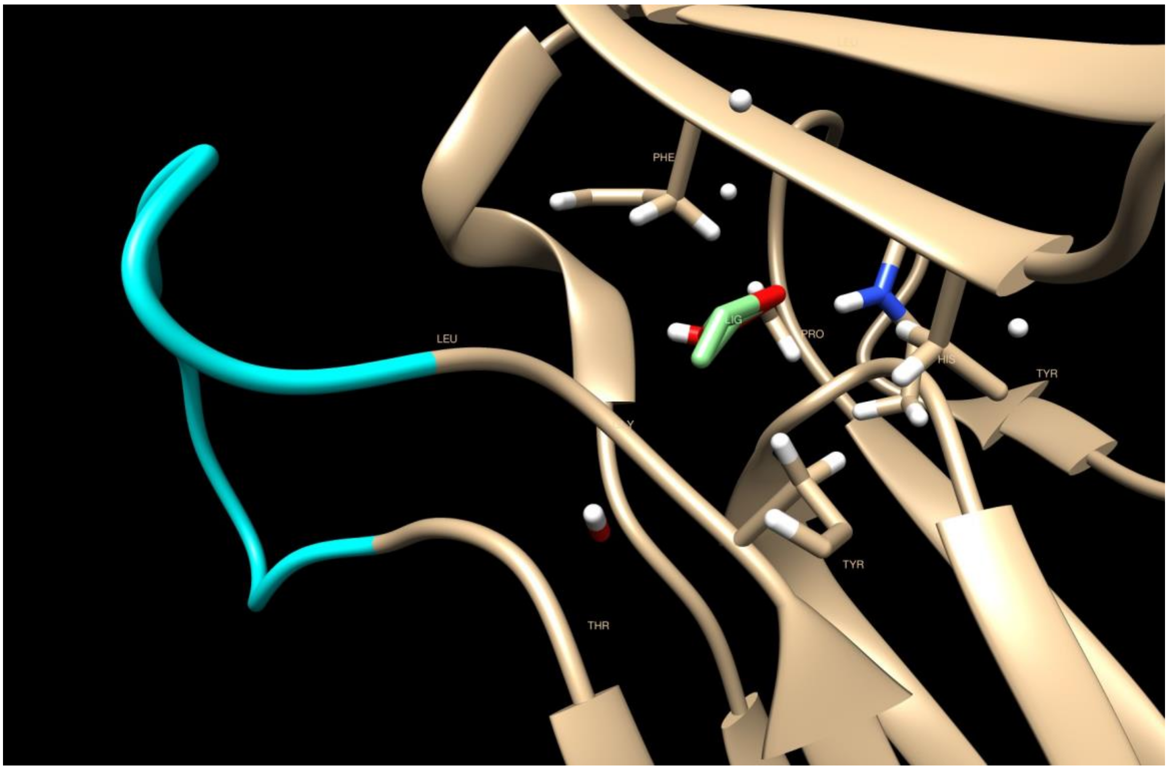

Propanoic acid, showing no H-bond to FG loop residues.

Supplementary Figure 3.

Ligand			
estradiol	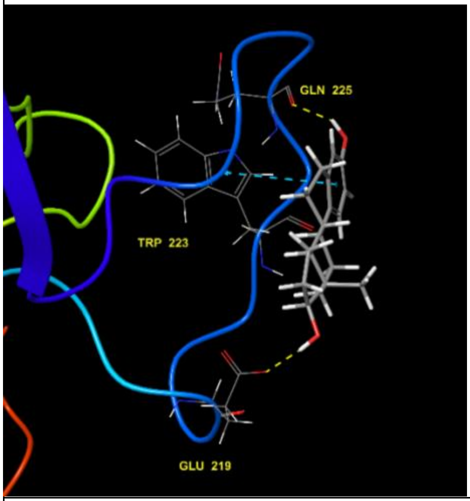		
	-31.3		
sucrose	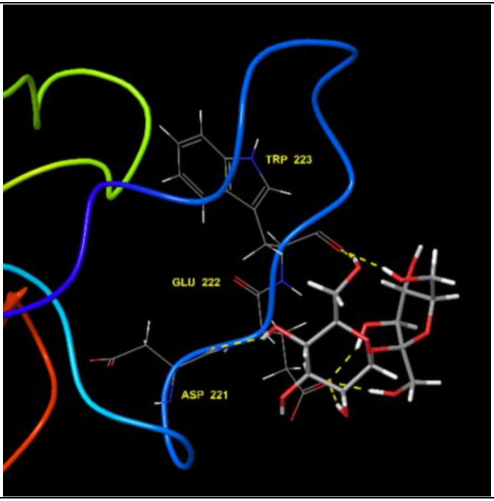		
	-29.7		

aspartame	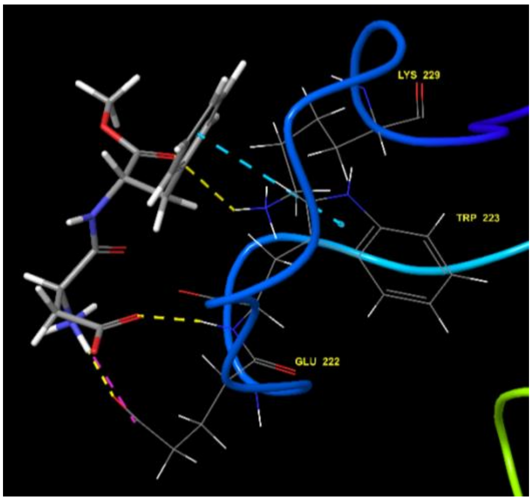	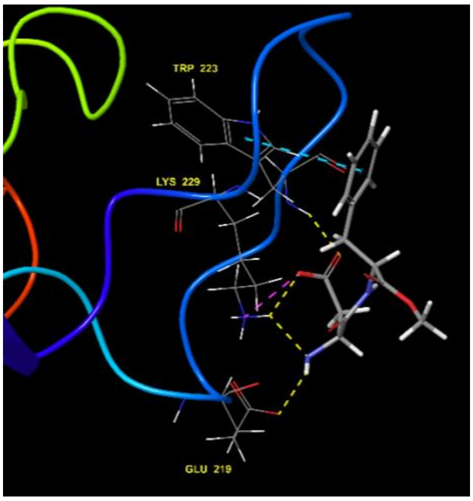	
	-27.9	-16.7	
butanediol	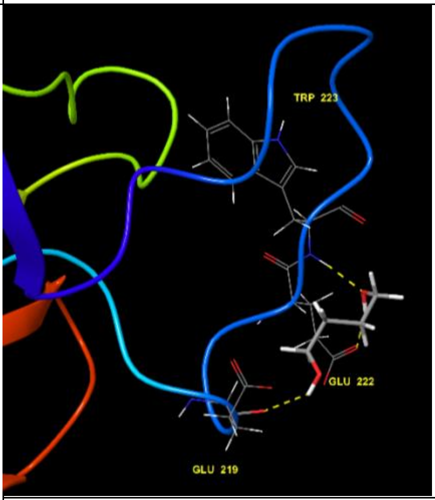		
	-21.1		
indolebutyrate	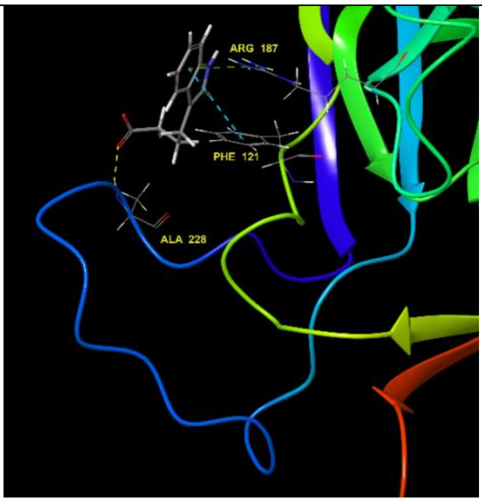		
	-20.9		

nicotine	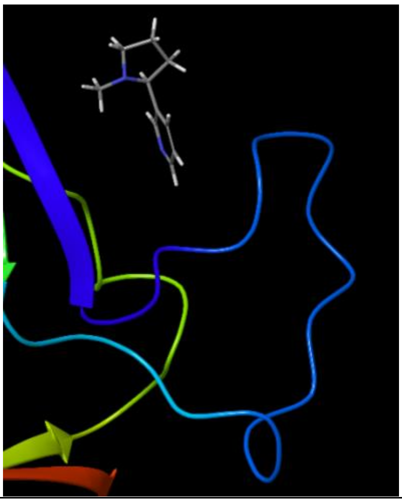	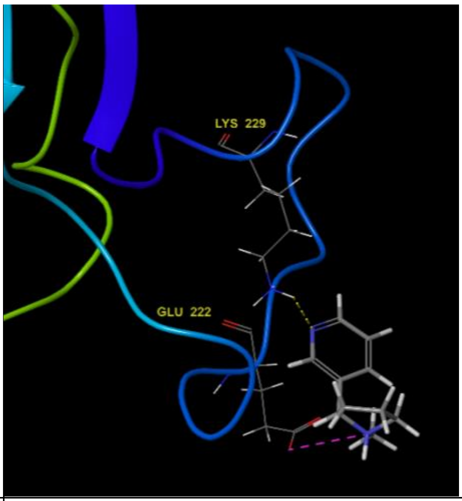	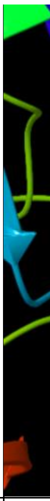
	-20.6	-15.3	
homotryptophan	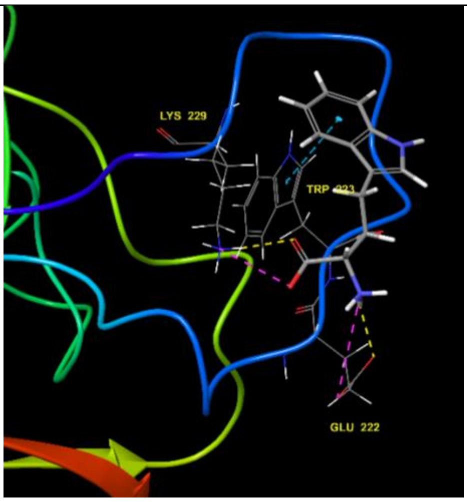	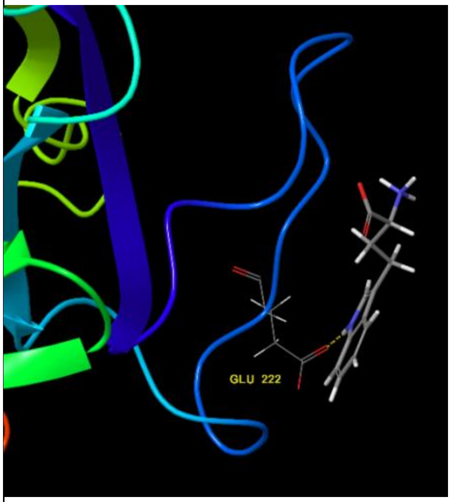	
	-18.3	-17.2	
NAD_tryptophan	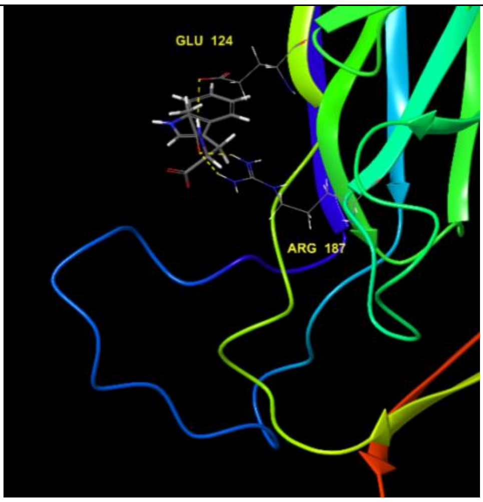		
	-17.3		

glycerol	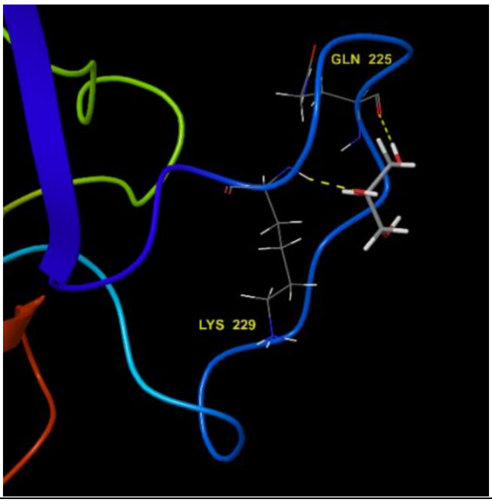		
	-13.8		
malonic	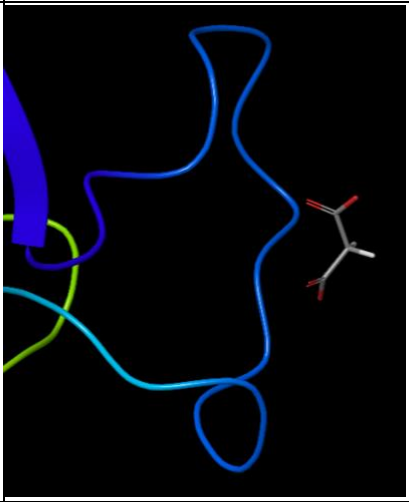	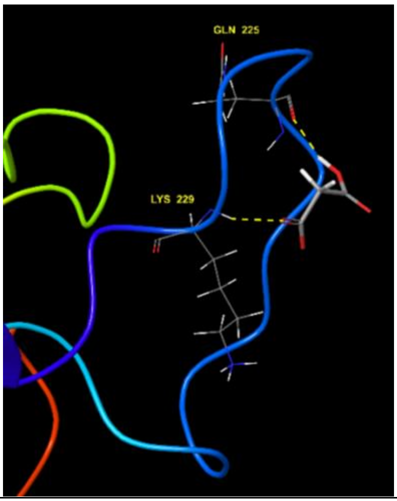	
	-9.7	0.6	
propanoic	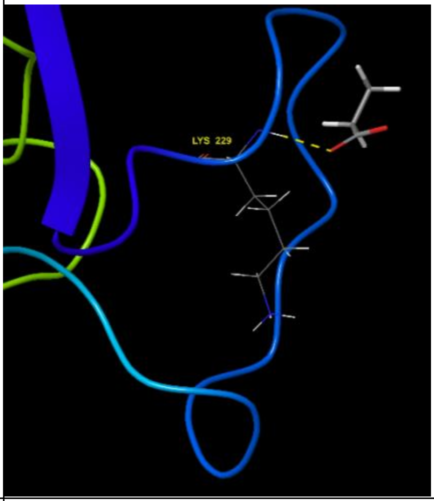		
	-5.5		

isobutyrate	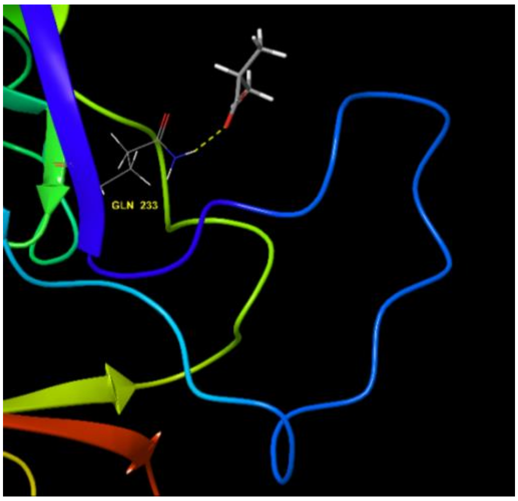		
	-4.5		

Supplementary Figure 4

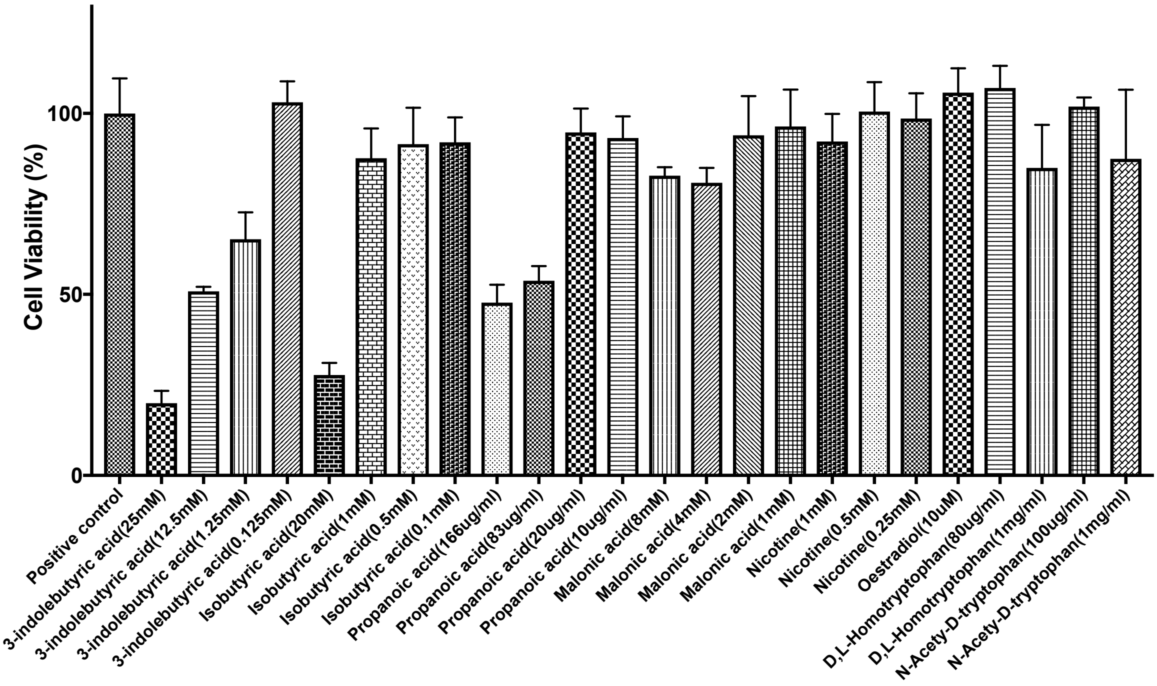

Supplement: Supplementary file 1 [file ijms-24-09334-s001.zip › Supplementary Figures.pdf]
