# Supplementary material for: Non-Antigenic Modulation of Antigen Receptor (TCR) Cβ-FG Loop Modulates Signalling: Implications of External Factors Influencing T-Cell Responses
Source: Int J Mol Sci. 2023 May 26;24(11):9334. doi: 10.3390/ijms24119334 (PMC10253556; doi:10.3390/ijms24119334)
Supplement: Supplementary file 1 [file ijms-24-09334-s001.zip › Supplementary methodology.pdf]

Supplementary online methodology

Preparation of protein structure

The 3-D crystal structure of TCR (ID: 1OGA, 1.4Å resolution) was retrieved from the protein data bank (PDB; www.rcsb.org/pdb) [18]. Structural domain analysis of the proteins was performed using UCSF Chimera and PyMol. The structure was subsequently saved in PDBQT file format that contains a protein structure with hydrogen in all polar residues.

Molecular docking

The docking sites of TCR were analysed by PyRx and UCSF Chimera, which are widely used Computational Drug Discovery (CDD) software for screening libraries of compounds against potential drug targets. Both software use AutoDock to perform the docking of ligand to a set of grids (pre-calculated by AutoGrid). The generated list of target protein is based on Lamarckian genetic algorithm and empirical free energy scoring function:

$$\Delta G = \left(V_{bound}^{L-L} - V_{unbound}^{L-L}\right) + \left(V_{bound}^{P-P} - V_{unbound}^{P-P}\right) + \left(V_{bound}^{P-L} - V_{unbound}^{P-L} + \Delta S_{conf}\right)$$

$$V = W_{vdw} \sum_{i,j} \left(\frac{A_{ij}}{r_{ij}^{12}} - \frac{B_{ij}}{r_{ij}^{6}}\right) + W_{hbond} \sum_{i,j} E(t) \left(\frac{C_{ij}}{r_{ij}^{12}} - \frac{D_{ij}}{r_{ij}^{10}}\right) + W_{elec} \sum_{i,j} \frac{q_i q_j}{e(r_{ij}) r_{ij}} + W_{sol} \sum_{i,j} (S_i V_j + S_j V_i)\, e^{(-r_{ij}^2/2\sigma^2)}$$

Screening of compound libraries and active site validation

Seven compound libraries were downloaded from the respective databases detailed in Table 1. All libraries were screened against the TCR and its isolated Cβ domain using two approaches independently. In the first approach, individual ligands to TCR were geometrically optimised and docked using PyRx. The screening results based on binding affinities were then ranked and the top 100 compounds were considered as 'hits' in this study. In the second approach, all seven libraries were screened using FINDSITE (CSSB, Georgia Tech), which predicts ligand-binding pockets based on the binding site similarity among superimposed groups of template structures identified from threading. The top compounds between two approaches were compared and generated a list of top 100 compounds (Appendix 1). After the top compounds were identified and validated using cytokine assay, they were re-docked to TCR using Chimera. The structure was saved as .PDB file and further explored to predict the binding sites using "Ligand Explorer" software.

The ligand-binding site represents the site where the ligands most efficiently bind to TCR region of interest.

Target protein preparation

The X-ray crystal structure of the T-cell receptor (1OGA) was retrieved from the RCSB Protein Data Bank (https://www.rcsb.org/) . The protein structure was prepared using preparation and refinement protocols, directed by the Protein Preparation Wizard embedded in Maestro v11.8 (Schrödinger, LLC, New York, USA). This process includes assigning bond orders, adding hydrogen atoms, creating zero-order bonds to metals and disulphide bonds, filling in missing side chains using Prime v5.4 and deleting water molecules beyond 5 Å from heteroatoms. The hydrogen bond network within the protein was also optimised and the protein structure minimised to a root mean square deviation (RMSD) of 0.3 Å using the OPLS3force field,

Ligand preparation

The ligands used include aspartame, estradiol, glycerol, homotryptophan, nicotine, oxaliplatin, sucrose, butanediol, NAD-tryptophan, indole, isobutyric acid, malonate and proponoate. All ligands were prepared using the LigPrep v4.8 module to generate possible stereoisomers of the ligands as well as generating all potential ionisation states at pH 7±2.

Receptor grid generation

The Receptor Grid Generation tool in Glide v8.1 was used to characterise the binding site used for the docking studies. The binding sites was defined by a 20 $Å^3$ bounding box centred at the centroid of the residues 218-232 of Chain E of the protein. This encompasses the whole FG loop. A Coulomb-van der Waals scaling factor of 1.0 for receptor van der Waals radii was applied to protein atoms with a partial charge of less than 0.25. A similar factor of 0.8 was applied to ligand atoms with a partial charge cutoff of 0.15 e. Rotations of hydroxyl and thiol groups were not allowed.

Docking studies

The prepared ligands were docked into the receptor grids with Glide v8.1. All docking was carried out using the Extra Precision (XP) scoring function to refine binding energy estimates. All ligands were docked with flexible states to allow sampling the effect of nitrogen inversion and changing ring conformations.

Preparation for cytokine assays

All materials provided for the cytokine assays were listed in Table 2. For the cytokine assay, 2B4.11 cell line and B cell hybridoma line LK35.2 (LK, I-E^k bearing) were used. The 2B4.11 cell line is a murine T-cell hybridoma that expressed a complete TCR on the cell surface that specifically recognised and produced cytokines in response to recognition of pigeon breast muscle (PCC) antigen. The LK35.2 (LK, I-E^k bearing) line presented as the antigen-presenting cell. Both 2B4.11 and LK35.2 cells were cultured in RPMI (Roswell Park Memorial Institute) media supplemented with 10 % heat-inactivated fetal calf serum, 4 mM glutamine and 100 U/ml penicillin/streptomycin at 37 °C / 5 % CO_2.

Cell viability assay

2B4.11 ($n = 2.5\times10^4$) and LK35.2 ($n = 2.5\times10^4$) cells were incubated in the presence or absence of compounds of interest. After 24 hours (h), cell viability was assessed by haemocytometer with 2.0% Trypan-blue. The following equation was used to generate the percentage of cell viability :

$$\text{Number of viable cells} = \frac{Q1+Q2+Q3+Q4}{4} \times 10^4 \times 2 \times sample\ dilution$$

$$\text{Percentage of viable cells} = \frac{Number\ of\ viable\ cells}{Total\ number\ of\ cells}$$

Antigen presentation assay

The assay was described previously to assess T-cell cytokines produced in response to antigen [31]. Briefly, 2B4.11 ($n = 2.5\times10^4$) cells and LK35.2 ($n = 2.5\times10^4$) cells were incubated with PCC (50 μM) in a 96-well micro-plate in the presence or absence of compounds. After 16 h, the plates were centrifuged at 2,000 rpm for 5 min. IL-2, IL-6, IFN-γ, TGF-β and GM-CSF levels in the supernatant were measured with an ELISA kit according to the manufacturers' instructions.

Statistical analysis

For cytokine assay, the normalised cytokine productions (ng/mL) between compounds were compared using ANOVA and Dunnett's multiple comparison tests, implemented in GraphPad Prism 6.0. Statistical significance was defined as $p < 0.05$.
