# Supplementary material for: Non-Antigenic Modulation of Antigen Receptor (TCR) Cβ-FG Loop Modulates Signalling: Implications of External Factors Influencing T-Cell Responses
Source: Int J Mol Sci. 2023 May 26;24(11):9334. doi: 10.3390/ijms24119334 (PMC10253556; doi:10.3390/ijms24119334)
Supplement: Supplementary file 1 [file ijms-24-09334-s001.zip › Supplementary Tables.pdf]

Supplementary Table 1

Table 1. The list of compound libraries and databases.

Compound library	Database website	Origin
BindingDB	https://www.bindingdb.org/bind/index.jsp	UCSD, USA
ChEMBL model	https://www.ebi.ac.uk/chembl/target	EBI, UK
Drug bank	https://www.drugbank.ca/	Alberta, Canada
KEGG compound	http://www.genome.jp/kegg/compound/	Kyoto, Japan
KEGG drug	http://www.genome.jp/kegg/drug/	Kyoto, Japan
NCI open database	https://cactus.nci.nih.gov/download/nci/	NIH, USA
ZINC 8	https://zinc8.docking.org/	UCSF, USA

Supplementary Table 2.

List of 100 final ranked compounds from seven screened public libraries.

ID	Name	Database
C11284	Indolebutyric acid / Indole-3-butyric acid	KEGG compounds
DB02740	3-Indolebutyric	ZINC8DB
NSC_003130	N-[3-bromophenyl]-1-[4-chlorophenyl]-6-methyl-1H-pyrazolo[3,4-d]pyrimidin-4-amine	NCI open cactus
ZINC00057378	Indole-3-butyric acid	12M
NSC_520394	methyl 4-[1H-indol-3-yl]butanoate	NCI open cactus
ZINC00410211	Methyl 4-[1H-Indol-3-yl]butanoate	12M
C00116	Glycerol;Glycerin;1,2,3-Trihydroxypropane;1,2,3-Propanetriol	KEGG compounds
C02773	1-Alkyl-sn-glycerol; 1-O-Alkyl-sn-glycerol	KEGG compounds
D00028	COCA-GLYCERINE CONTROL [Antigen Laboratories]	KEGG Drugs
D05073	Monoctanoin [USAN]; Moctanin [TN]	KEGG Drugs
DB04077	Glycerol	ZINC8DB
NSC_009230	glycerol	NCI open cactus
NSC_403834	2-methyl-1,2,3-propanetriol	NCI open cactus
ZINC37632578	5-[1H-indol-3-yl]pentanoic	12M
NSC_615268	C38H69N13O14	NCI open cactus
ZINC02566960	6-[1H-indol-3-yl]hexanoic acid	12M
ZINC37632580	7-[1H-indol-3-yl]heptanoic	12M
ZINC37632582	9-[1H-indol-3-yl]nonanoic	12M
DB02418	[R,R]-2,3-Butanediol	ZINC8DB
L002272	C40 H73 N11 O12 S	GLIDA ZINC 8
NSC_158562	ethyl 4-[1H-indol-3-yl]butanoate	NCI open cactus

NSC_520393	isopropyl 4-[1H-indol-3-yl]butanoate	NCI open cactus
ZINC00259399	ethyl 4-[1H-indol-3-yl]butanoate	12M
C03137	N-Acetyl-D-tryptophan	KEGG Compound
ZINC47311811	propyl 4-[1H-indol-3-yl]butanoate	12M
BDB0003879	[2S]-2-[[[2S]-1-[[2S]-2-[[[2S,3S]-2-[[[2S]-2-[[[2S]-2-acetamido-4-hydroxy-4-oxo-butanoyl]amino]-4-hy	Zinc 26376588
DB02531	Isobutyric	Zinc 8
DB03766	Propanoic	Zinc 8
C02170	Methylmalonate; Methylmalonic acid	KEGG Compound
C06388	2-Aryl-2-methylmalonate	KEGG Compound
DB02175	Malonic	Zinc 8
DB02201	Malonate	Zinc 8
DB04183	Methylmalonic	Zinc 8
NSC_000836	Dimethylmalonic acid; Malonic acid, dimethyl-2,2-Propanedicarboxylic acid	NCI open cactus
NSC_025201	Isosuccinic acid; Malonic acid, methyl-Methylmalonic acid; Propanedioic acid, methyl-1, 1-Ethanedicarboxylic acid	NCI open cactus
NSC_120443	Methanetricarboxylic acid	NCI open cactus
NSC_154847	cis-Diammine[methylmalonato]platinum[II]	NCI open cactus
NSC_154848	Platinum, [ethylenediamino][methylmalonato]-	NCI open cactus
NSC_175779	CC[C][C[O]=O]C[=O][OH+][Co+3][N][N][N][N]N.[O-][Cl][=O][=O]=O	NCI open cactus
NSC_215156	cis-Diammine[methylmalonato]platinum[II]	NCI open cactus
ZINC02566035	D,L-Homotryptophan	12M

ZINC04240327	[S]-4-[INDOL-3-YL]-BETA-HOMOALANINE HYDROCHLORIDE	12M
ZINC04521117	[R]-3-Amino-4-[1H-indol-3-yl]butanoic acid hydrochloride	12M
ZINC06864822	D,L-Homotryptophan	12M
C01083	alpha,alpha-Trehalose; alpha,alpha'-Trehalose; Trehalose	KEGG Compound
C14689	Diethylene glycol; 2,2'-Oxydiethanol; 1,5-Dihydroxy-3-oxapentane	KEGG Compound
NSC_036391	Diethylene glycol	NCI open cactus
ZINC05082162	2-[hydroxymethyl]-5-[1,2,4-triazol-1-yl]tetrahydrofuran-3,4-diol	Zinc 8
NSC_631177	29-amino-2,23-bis[4-aminobutyl]-26-sec-butyl-11-[carboxymethyl]-14,17-bis[1-hydroxyethyl]-8-isobutyl-4,7,10,13,16,19,22,25,28-nonaoxo-3,6,9,12,15,18,21,24,27-nonaazadotriacontane-1,32-dioic acid [ACD/Name 4.0]	NCI open cactus
L002222	L002222	Glida Zinc 8
D08904	Diethylene glycol monoethyl ether [NF]	KEGG Drug
DB02327	2-[2-[2-Hydroxy-Ethoxy]-Ethoxy]-Ethanol	Zinc 8
DB02343	3,6,9,12,15-Pentaoxaheptadecane	Zinc 8
DB02580	1-[2-Methoxy-Ethoxy]-2-[2-[2-[2-Methoxy-Ethoxy]-Ethoxy]-Ethane	Zinc 8
DB02935	1-Methoxy-2-[2-Methoxyethoxy]Ethane	Zinc 8
DB03508	1-Ethoxy-2-[2-Methoxyethoxy]Ethane	Zinc 8
DB03556	2-[2-[2-[2-[2-[2-[2-[2-Ethoxy-Ethoxy]-Ethoxy]-Ethoxy}-Ethoxy]-Ethoxy]-Ethoxy}-Ethoxy]-Ethanol,	Zinc 8
DB04332	2-[2-[2-2-[Methoxy-Ethoxy]-Ethoxy]-Ethoxy]-Ethanol	Zinc 8
DB06867	3,6,9,12,15,18-HEXAOXAICOSANE	Zinc 8
DB07344	3,6,9,12,15-PENTAOXAHEPTADECAN-1-OL	Zinc 8
DB08357	1-ETHOXY-2-[2-ETHOXYETHOXY]ETHANE	Zinc 8

NSC_363142	tetrabromostannane compound with 1,4,7,10-tetraoxacyclododecane [1:1] [ACD/Name 4.0]	NCI open cactus
NSC_363143	Stannane, dichlorodiphenyl-, compd. with 1,4,7,10,13, 16-hexaoxacyclooctadecane [1:1]	NCI open cactus
NSC_363144	Stannane, dichlorodimethyl-, compd. with 1,4,7,10,13, 16-hexaoxacyclooctadecane [1:1]	NCI open cactus
NSC_408451	Carbitol	NCI open cactus
NSC_631895	18-Crown-6-tetrachloroplatinum[IV]	NCI open cactus
NSC_632590	18-Crown-6-tantalum[V]pentachloride	NCI open cactus
NSC_632592	18-Crown-6-tantalum[V]pentachloride	NCI open cactus
ZINC01690436	2,2'-[Ethane-1,2-diylbis[oxy]]diethanol	Zinc 8
C00089	sucrose	KEGG Compound
C04219	alpha-D-Aldosyl beta-D-fructoside; alpha-D-Aldosyl1 beta-D-fructoside; alpha-D-Aldosyl2 beta-D-fructoside	KEGG Compound
C07314	Sucralfate	KEGG Compound
D00025	Suc	KEGG Drug
D00446	Sucralfate [USP/INN]	KEGG Drug
L000836	Cocaine	Glida Zinc 8
BDB0017292	Estradiol	zinc13520815
BDB0017294	[E]-3-cyclopropyl-N-methyl-3-[[2R]-oxiran-2-yl]prop-2-en-1-amine	zinc14963904
BDB0020624	Estradiol	Zinc 3815415
BDB0022422	Estradiol	Zinc 13420815
BDB0023952	Estradiol	Zinc 13420815

BDB0025868	Estradiol	Zinc 13420815
C00951	Estradiol-17beta; Estradiol; beta-Estradiol	KEGG Compound
C02537	Estradiol-17alpha; 17alpha-Estradiol	KEGG Compound
C14483	17alpha-Methylestradiol	KEGG Compound
C15129	Estra-1,3,5[10]-triene-2,17beta-diol; 2-Hydroxy-3-deoxyestradiol	KEGG Compound
C15261	Estra-1,3,5[10]-triene-3,16beta-diol; 16beta-Estradiol	KEGG Compound
D00105	Sucrose	KEGG Drug
D07121	Alfatradiol [INN] 17alpha-Estradiol	KEGG Drug
L024123	Estradiol	Glida Zinc 8
C11045	Aspartame	KEGG Compound
D02381	Aspartame [NF/INN]	KEGG Drug
D08862	Aspartame acesulfame [NF]	KEGG Drug
L001103	Aspartame	Glida Zinc 8
BDB0010357	[4S]-4-[[[2S,3S]-2-acetamido-3-methyl-pentanoyl]amino]-5-[[[1S,2S]-1-[[[1S]-1-formyl-3-hydroxy-3-oxo	Zinc14947180
C00745	Nicotine; [S]-3-[1-Methylpyrrolidin-2-yl]pyridine; [S]-Nicotine	KEGG Compound
C16150	R,S]-Nicotine; [+-]-3-[1-Methyl-2-pyrrolidinyl]pyridine	KEGG Compound
C16386	[R]-Nicotine; D-Nicotine; 2'-beta-H-Nicotine	KEGG Compound
D03365	Nicotine [USP]	KEGG Drug
D05156	Nicotine bitartrate [USAN]	KEGG Drug

Supplementary Table 3

Statistical analysis of cytokine assay results

Dunnett's multiple comparisons test	Mean Diff.	95.00 CI of diff.	Summary	Adjusted P Value	
**IL2**					
Positive vs. Oestradiol(10uM)	628.9	560 to 697.8	****	0.0001	Oestradiol(10uM)
Positive vs. 3-indolebutyric acid (0.5mM)	49	-19.86 to 117.9	ns	0.2784	3-indolebutyric acid (0.5mM)
Positive vs. Aspartame(1mg/ml)	96.1	27.24 to 165	**	0.0028	Aspartame(1mg/ml)
Positive vs. Butanediol (0.175%)	92.3	23.44 to 161.2	**	0.0043	Butanediol (0.175%)
Positive vs. D,L-Homotryptophan (1mg/ml)	316.5	247.6 to 385.4	****	0.0001	D,L-Homotryptophan (1mg/ml)
Positive vs. Glycerol (1mM)	104.8	35.94 to 173.7	**	0.001	Glycerol (1mM)
Positive vs. Isobutyric acid (0.5mM)	40.9	-27.96 to 109.8	ns	0.4802	Isobutyric acid (0.5mM)
Positive vs. Malonic acid (1mM)	47.5	-21.36 to 116.4	ns	0.3103	Malonic acid (1mM)
Positive vs. N-Acety-D-tryptophan (1mg/ml)	118.2	49.34 to 187.1	***	0.0002	N-Acety-D-tryptophan (1mg/ml)
Positive vs. Nicotine (0.5mM)	91.9	23.04 to 160.8	**	0.0045	Nicotine (0.5mM)
**Positive vs. Oxaliplatin(1ng/ml)**	**-93.1**	**-164.9 to -21.32**	**	**0.0059**	**Oxaliplatin(1ng/ml)**
Positive vs. Propanoic acid (10ug/ml)	43.3	-25.56 to 112.2	ns	0.4133	Propanoic acid (10ug/ml)
Positive vs. Sucrose (0.01%)	83.8	14.94 to 152.7	*	0.0108	Sucrose (0.01%)
**IL6**					
Positive vs. 3-indolebutyric acid (0.5mM)	-33.3	-96.4 to 29.8	ns	0.6035	3-indolebutyric acid (0.5mM)
Positive vs. Aspartame(1mg/ml)	-20.1	-83.2 to 43	ns	0.958	Aspartame(1mg/ml)
Positive vs. Butanediol (0.175%)	11.7	-51.4 to 74.8	ns	0.9991	Butanediol (0.175%)
Positive vs. D,L-Homotryptophan (1mg/ml)	-29.5	-92.6 to 33.6	ns	0.7302	D,L-Homotryptophan (1mg/ml)
Positive vs. Glycerol (1mM)	26.7	-36.4 to 89.8	ns	0.817	Glycerol (1mM)
Positive vs. Isobutyric acid (0.5mM)	16.7	-46.4 to 79.8	ns	0.9886	Isobutyric acid (0.5mM)
Positive vs. Malonic acid (1mM)	-55	-118.1 to 8.1	ns	0.1119	Malonic acid (1mM)
Positive vs. N-Acety-D-tryptophan (1mg/ml)	-24.1	-87.2 to 39	ns	0.8852	N-Acety-D-tryptophan (1mg/ml)
Positive vs. Nicotine (0.5mM)	-11.6	-74.7 to 51.5	ns	0.9992	Nicotine (0.5mM)
Positive vs. Oxaliplatin(1ng/ml)	3.4	-59.7 to 66.5	ns	0.9998	Oxaliplatin(1ng/ml)

Positive vs. Propanoic acid (10ug/ml)	15	-48.1 to 78.1	ns	0.9923	Propanoic acid (10ug/ml)
Positive vs. Sucrose (0.01%)	23.4	-39.7 to 86.5	ns	0.9009	Sucrose (0.01%)
**IL10**					
Positive vs. 3-indolebutyric acid (0.5mM)	-38.3	-128.3 to 51.67	ns	0.8122	3-indolebutyric acid (0.5mM)
Positive vs. Aspartame(1mg/ml)	-4.5	-94.47 to 85.47	ns	0.9998	Aspartame(1mg/ml)
Positive vs. Butanediol (0.175%)	10.8	-79.17 to 100.8	ns	0.9995	Butanediol (0.175%)
Positive vs. D,L-Homotryptophan (1mg/ml)	-89.5	-179.5 to 0.4717	ns	0.0517	D,L-Homotryptophan (1mg/ml)
Positive vs. Glycerol (1mM)	10.8	-79.17 to 100.8	ns	0.9995	Glycerol (1mM)
Positive vs. Isobutyric acid (0.5mM)	-23.3	-113.3 to 66.67	ns	0.9899	Isobutyric acid (0.5mM)
Positive vs. Malonic acid (1mM)	-10	-99.97 to 79.97	ns	0.9996	Malonic acid (1mM)
Positive vs. N-Acety-D-tryptophan (1mg/ml)	-43.8	-133.8 to 46.17	ns	0.6899	N-Acety-D-tryptophan (1mg/ml)
Positive vs. Nicotine (0.5mM)	10.8	-79.17 to 100.8	ns	0.9995	Nicotine (0.5mM)
Positive vs. Oxaliplatin(1ng/ml)	10.8	-79.17 to 100.8	ns	0.9995	Oxaliplatin(1ng/ml)
Positive vs. Propanoic acid (10ug/ml)	-18.3	-108.3 to 71.67	ns	0.9966	Propanoic acid (10ug/ml)
Positive vs. Sucrose (0.01%)	10.8	-79.17 to 100.8	ns	0.9995	Sucrose (0.01%)
**IFN gamma**					
Positive vs. 3-indolebutyric acid (0.5mM)	-0.6	-70.62 to 69.42	ns	0.9999	3-indolebutyric acid (0.5mM)
Positive vs. Aspartame(1mg/ml)	-34	-104 to 36.02	ns	0.6926	Aspartame(1mg/ml)
Positive vs. Butanediol (0.175%)	27.9	-42.12 to 97.92	ns	0.8595	Butanediol (0.175%)
Positive vs. D,L-Homotryptophan (1mg/ml)	34.7	-35.32 to 104.7	ns	0.6716	D,L-Homotryptophan (1mg/ml)
Positive vs. Glycerol (1mM)	-0.9	-70.92 to 69.12	ns	0.9999	Glycerol (1mM)
Positive vs. Isobutyric acid (0.5mM)	0.9	-69.12 to 70.92	ns	0.9999	Isobutyric acid (0.5mM)
Positive vs. Malonic acid (1mM)	-10.9	-80.92 to 59.12	ns	0.9993	Malonic acid (1mM)
**Positive vs. N-Acety-D-tryptophan (1mg/ml)**	**-73.9**	**-143.9 to -3.877**	*	**0.0346**	**N-Acety-D-tryptophan (1mg/ml)**
Positive vs. Nicotine (0.5mM)	-27.8	-97.82 to 42.22	ns	0.8619	Nicotine (0.5mM)

Positive vs. Oxaliplatin(1ng/ml)	4.9	-65.12 to 74.92	ns	0.9997	Oxaliplatin(1ng/ml)
Positive vs. Propanoic acid (10ug/ml)	10	-60.02 to 80.02	ns	0.9994	Propanoic acid (10ug/ml)
Positive vs. Sucrose (0.01%)	4.3	-65.72 to 74.32	ns	0.9997	Sucrose (0.01%)

Supplementary Table 4

Ranking of active compounds on specific cytokine assay in order with strongest cytokine production first

Compounds	95.00% CI of diff.	Summary	Adjusted P Value	
Oestradiol(10uM)	560 to 697.8	****	0.0001	Oestradiol(10uM)
D,L-Homotryptophan (1mg/ml)	247.6 to 385.4	****	0.0001	D,L-Homotryptophan (1mg/ml)
N-Acety-D-tryptophan (1mg/ml)	49.34 to 187.1	***	0.0002	N-Acety-D-tryptophan (1mg/ml)
Glycerol (1mM)	35.94 to 173.7	**	0.001	Glycerol (1mM)
Aspartame(1mg/ml)	27.24 to 165	**	0.0028	Aspartame(1mg/ml)
Oxaliplatin(1ng/ml)	-164.9 to -21.32	**	0.0059	Oxaliplatin(1ng/ml)
Butanediol (0.175%)	23.44 to 161.2	**	0.0043	Butanediol (0.175%)
Nicotine (0.5mM)	23.04 to 160.8	**	0.0045	Nicotine (0.5mM)
Sucrose (0.01%)	14.94 to 152.7	*	0.0108	Sucrose (0.01%)
TGF beta				
Butanediol (0.175%)	42.62 to 210.8	**	0.0013	Butanediol (0.175%)
Glycerol (1mM)	7.217 to 175.4	*	0.0281	Glycerol (1mM)
**Malonic acid (1mM)**	**-191.1 to -22.92**	**	**0.0075**	**Malonic acid (1mM)**
**N-Acety-D-tryptophan (1mg/ml)**	**-187.2 to -19.02**	*	**0.0105**	**N-Acety-D-tryptophan (1mg/m**
IL2 oxiplatin				
Oxaliplatin(1ng/ml)	-243.8 to -124.2	****	0.0001	Oxaliplatin(1ng/ml)
Oxaliplatin(5ng/ml)	-135.4 to -15.79	*	0.0144	Oxaliplatin(5ng/ml)
